# Efficacy of Arabic Coffee and Black Tea in Reducing Halitosis: A Randomized, Double-Blind, Controlled, Crossover Clinical Trial

**DOI:** 10.3390/healthcare9030250

**Published:** 2021-03-01

**Authors:** Hamad Alzoman, Ahmed Alzahrani, Khaled Alwehaiby, Waleed Alanazi, Mohammed AlSarhan

**Affiliations:** 1 Department of Periodontics and Community Dentistry, College of Dentistry, King Saud University, Riyadh 11545, Saudi Arabia; malsarhan@ksu.edu.sa; 2 Dental Intern, College of Dentistry, King Saud University, Riyadh 11545, Saudi Arabia; Drahmediz93@gmail.com (A.A.); K.Alwehaiby@gmail.com (K.A.); waleedk.alanazi@gmail.com (W.A.)

**Keywords:** Arabic coffee, black tea, *Camellia sinensis*, cysteine challenge, oral malodor, cardamom

## Abstract

The aim of the study was to objectively evaluate the short-term effect of Arabic coffee and black tea on oral halitosis. This study was a single-center, randomized, double-blind, placebo-controlled, crossover clinical trial on 17 healthy individuals. During the initial visit, pre-treatment breath samples were collected from each subject and analyzed using portable gas chromatography (OralChroma™). Four interventions were evaluated, with Arabic coffee and black tea as the test intervention tools, mouthwash containing a solution (0.05% chlorhexidine, 0.05% cetylpyridinium chloride, and 0.14% zinc lactate (CHX-CPC-Zn)) as a positive control, and drinking water as a negative control. Halitosis was induced by rinsing with 10 mL solution of L-cysteine for 30 s. Twenty minutes later, a breath sample was taken to record the baseline volatile sulfur compounds (VSC) levels (T0). Then, the participants were asked to rinse with 10 mL of a randomly-assigned solution for 30 s. Sixty minutes later, another breath sample was recorded (T1). Finally, after 120 min, the final breath sample was recorded (T2). It was found that rinsing with Arabic coffee decreased the level of H_2_S both in the first hour (T1) and the second hour (T2). The reduction was significantly greater at T1 (*p* = 0.017). There was a similar result after the volunteers rinsed with black tea. At T2, Arabic coffee showed a substantially greater reduction in H_2_S (*p* < 0.001). On the contrary, using CHX-CPC-Zn showed a significant and continuous decrease in H_2_S values in the breath throughout the experiment (*p* < 0.001). Water showed no significant impact on the level of VSC (*p* = 0.71). This study demonstrates that black tea and Arabic coffee had inhibitory effects on halitosis that was greater in the first hour and was not sustained over a long period. Additionally, Arabic coffee had a greater inhibitory effect on halitosis than black tea.

## 1. Introduction

Halitosis is a term of Latin origin derived from halitus (breathed air) and osis (pathologic alteration). According to the Glossary of Periodontal Terms (2001), halitosis is defined as “breath that is offensive to others, caused by a variety of reasons, including, but not limited to, periodontal disease, bacterial coating of the tongue, systemic disorders, and different types of food” [1]. Various factors cause oral malodor, including those originating from the oral cavity, such as tongue coating, periodontal disease, peri-implant disease, deep carious lesions, exposed necrotic tooth pulps, pericoronitis, imperfect dental restorations, and food impaction [2,3,4]. On the contrary, non-oral halitosis results from upper or lower respiratory tract diseases, gastrointestinal tract disorders, metabolic disorders, some systemic diseases, and carcinomas [5].

Several odoriferous components lead to oral malodor. Volatile sulfur compounds (VSC) such as hydrogen sulfide (H_2_S), methyl mercaptan (CH3SH, MM), and dimethyl sulfide (CH3SCH3) were found to have a notable connection with oral malodor [6]. The bacteria produce odorous volatile sulfur compounds in the oral cavity [7].

The first line of therapy to treat halitosis should be to eliminate the identified causal factors. Reduced plaque accumulation could be achieved by mechanical and chemical methods or a combination of both [8]. Several types of mouthwash reportedly treat halitosis effectively with specific active ingredients, such as: chlorhexidine gluconate [9], essential oils [10], and triclosan [11]. Some reports suggest that mouthwashes consisting of chlorhexidine gluconate and a combination of cetylpyridinum chloride with zinc yield better outcomes in treating oral malodor [12,13]. Additionally, patients suffering from halitosis have used some natural herbal products that might provide a masking effect against bad breath odor. A survey carried out on 481 dental students found that around 44% of males and 37% of females reported drinking black tea to mask halitosis [14].

Coffee, a commonly brewed beverage worldwide [15], comes from beans of the genus *Coffea*. Coffee contains caffeic acid, chlorogenic acid, and trigonelline, which have both antibacterial and antioxidant effects. Coffee extracts reportedly have antimicrobial activity in addition to the ability to inhibit the protease production of *Porphyromonas gingivalis* [16]. Furthermore, coffee extract reportedly significantly minimized the concentration of VSC by 85%, as well as VSC-producing bacteria in vitro [17].

Tea is made from plant leaves of *Camellia sinensis*, which is considered the second most consumed beverage globally [15]. Of the total tea consumption, 20% is green tea, while 78% is black tea [18]. The fermentation of green tea leaves achieves black tea production; this fermentation process reportedly reduces some of the tea’s beneficial chemical components [19,20,21]. Tea contains several polyphenolic compounds, such as flavonols and catechins. These compounds have demonstrated a wide variety of antioxidant and antibacterial activities [20,22]. Zeng et al. showed reduced levels of hydrogen sulfide and MM after using green tea extract [23]. Other in vitro studies reported the antimicrobial effect of green tea extract. In vivo observations by Lodhia et al. found a significant reduction in VSC immediately due to green tea usage [24]. Up to this date, there is little evidence of the short-term effect of using Arabic coffee and black tea on the VSC of subjects suffering from halitosis.

This study aimed to evaluate the short-term effect of Arabic coffee and black tea on halitosis.

## 2. Materials and Methods

### 2.1. Study Design and Population

**Figure 1:**
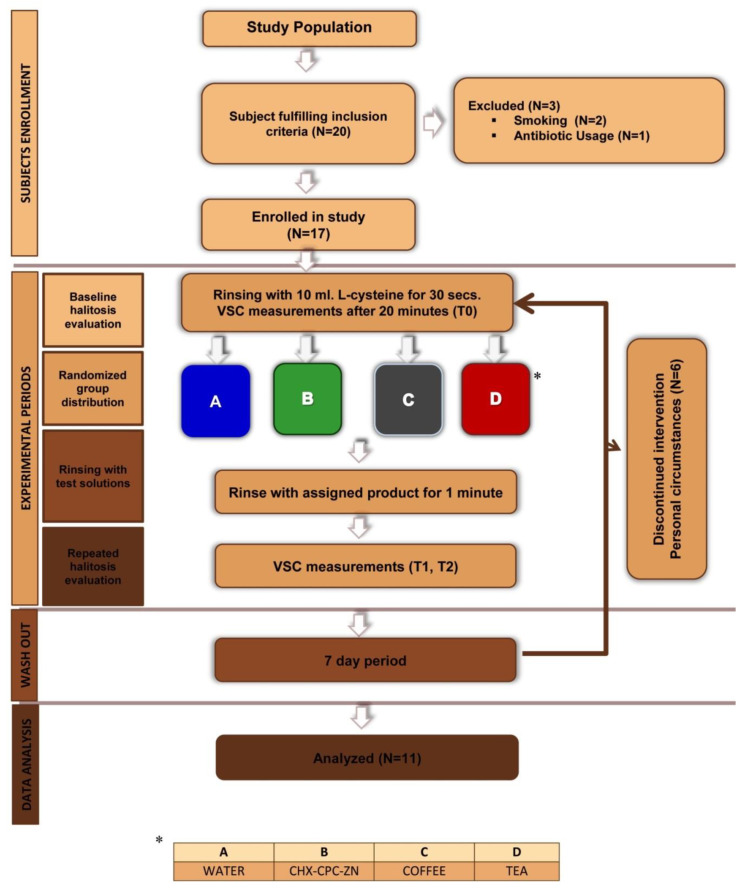
Consort flowchart of the study inclusion, allocation, and follow-up.

This study was a single-center, crossover, double-blind, randomized controlled clinical trial conducted at the College of Dentistry, King Saud University. The institutional review board of the King Saud University approved this study (No. 17/0316), and reported following the CONSORT 2010 guidelines (Figure 1).

All participants provided informed consent; 29 were initially screened for the following inclusion criteria:

1. Healthy male participants;
2. Age 18 years or older;
3. Absence of halitosis in exhaled air.

The participants were excluded from this study if they had any of the following conditions:

- Respiratory tract diseases;
- Tonsillitis;
- Stomach disorders;
- Antibiotic use in the previous three months;
- Being a smoker;
- Presence of fewer than 20 natural teeth.

The presence of halitosis was defined as the ability to detect volatile sulfur compounds (VSCs) in the breath sample equal to or greater than the cognitive threshold [Hydrogen Sulfide (H_2_S) ≥ 112 ppb or Methyl Mercaptan (CH3SH, MM) ≥ 26 ppb [25].

### 2.2. Study Protocol

The study enrolled 17 participants who fulfilled the inclusion criteria and consented to participate voluntarily. At the initial visit, a pre-treatment breath sample was collected from each subject. Additionally, the probing depth (PD), plaque index (PI) [26], and gingival index (GI) [27] were recorded.

All clinical measurements were performed by two examiners (AZ and KW). Calibration exercises for clinical measurements were performed in 10 patients not included in the study. The intra-examiner and inter-examiner reliability yielded kappa values of 0.9 and 0.88, respectively. At the end of the initial visit, each subject received an oral hygiene kit containing a dentifrice and a soft toothbrush, and they were instructed to use it throughout the study.

There were four interventions in this study: Arabic coffee and black tea used as test solutions, as well as mouthwash containing CHX-CPC-Zn (Halita^®^, Dentaid, Barcelona, Spain) as a positive control, and drinking water as a negative control.

Arabic coffee and black tea were standardized and freshly prepared daily. Black tea solution was prepared by adding 12 g of black tea (AlMunayes General Trading Est., Sharq, Kuwait) to a liter of boiled water. The tea was brewed for ten minutes.

The Arabic coffee solution prepared using 30 g of ground coffee (Jiad Al-Qassim, Al-Qassim, Saudi Arabia) was added to 800 mL of water and boiled for 15 min. Then 10 g of cardamom were added, and the solution was boiled for an additional 2 min, after which it was allowed to brew for 10 min. Later, the coffee solution was filtered to remove the coffee grounds. All solutions used in the four interventions were served in identical paper cups, covered with lids, and encoded as A, B, C, and D by another investigator (WA). To ensure the blindness of the study, neither participants nor examiners were aware of the codification. The subjects were randomly allocated to each intervention using a computer-generated randomization schedule.

### 2.3. Experimental Phase

At the beginning of each intervention, halitosis was induced by rinsing with 10 mL of 6 mM aqueous solution of L-cysteine solution for 30 s, according to the Kleinberg protocol [28]. Twenty minutes later, a breath sample was taken to record the increased VSC levels at baseline before the intervention (T0). After the baseline reading, the participant was asked to rinse with 10 mL of a randomly-allocated mouthwash for 30 s. Sixty minutes later, another breath sample was taken and recorded (T1). Finally, after 120 min, the final breath sample was taken (T2). The subjects were asked to refrain from eating, drinking, brushing, or using chewing gum throughout the experimental phase. The same procedure was repeated for the next intervention after one-week washout period (Figure 1).

### 2.4. VSC Assessment

VSC was detected using a portable Semiconductor Gas Sensor OralChroma^TM^ CHM-2 (Abilit, Osaka, Japan). Participants were requested to close their mouths and breathe with their noses for 60 s before the breath samples were collected. Then, using a disposable plastic syringe, a 1-mL breath sample was collected. Immediately, the collected breath sample was injected into the OralChroma device. The concentration of H_2_S and MM were recorded in ppb.

### 2.5. Statistical Tests

#### 2.5.1. Study Power

The sample size was determined using the Cohen (1988) procedure, by assuming an effect size (f) of 0.80 and with study power of 90% (1 − β = 0.90) and at α = 0.05. The study required at least 10 subjects to establish a statistically significant difference between the four study intervention groups. Since this was a crossover trial, more subjects were enrolled to guard against the possibility of withdrawal from follow-up.

#### 2.5.2. Statistical Analysis

Data were analyzed using the SPSS Pc+ version 21.0 statistical software. The descriptive statistics (mean and the standard deviation) were used to present the average value obtained for H_2_S and MM for each given intervention and time. A line graph was also used to present averages’ movement due to each intervention’s change in time. Furthermore, the difference in H_2_S and MM’s mean values due to variations in interventions at a given time (T0, T1, T2) was analyzed using the one-way ANOVA. Similarly, the effect of variation in time within each intervention was also studied for each gas through the one-way ANOVA. *p*-values less than 0.05 were considered statistically significant.

## 3. Results

Out of the 17 subjects, six withdrew from the study due to personal circumstances. Therefore, the data of 11 study subjects were analyzed. The age range was 20–24 years, with a mean age of 22 years (Table 1).

### 3.1. Impact of Arabic Coffee on Halitosis

The comparison of total VSC values between pre- and post-intervention using Arabic coffee showed statistically significant changes in the H_2_S values (*p* < 0.001), but there were no significant changes in those of MM (*p* = 0.005). At baseline, the means of H_2_S and MM were 446 ppb and 73 ppb, respectively. Two hours after treatment, they dropped to 230 ppb and 66 ppb, respectively.

### 3.2. Impact of Black Tea on Halitosis

Black tea demonstrated a significant impact on the VSC level. The baseline mean of H_2_S was 457 ppb. After treatment, it decreased to 307 ppb (*p* = 0.045). Meanwhile, the mean of MM at baseline was 101 ppb, which significantly reduced at 2 h to 79 ppb (*p* = 0.03) (Table 2).

### 3.3. Impact of CHX-CPC-Zn Mouthwash on Halitosis

At baseline, the mean level of H_2_S was 450 ppb. H_2_S concentration decreased to 74 ppb after 2 h of treatment with CHX-CPC-Zn (*p* < 0.001). Additionally, MM concentration decreased from 106 ppb to 36 ppb at 2 h after treatment (*p* = 0.005) Table 2.

### 3.4. Impact of Water on Halitosis

Water had the least impact on the VSC concentrations among all four interventions. At baseline, the means of H_2_S and MM were 412 ppb and 73 ppb, respectively. H_2_S reduced to 244 ppb, one hour after treatment, then increased, although not significantly, to 384 ppb (*p* = 0.71). Similarly, there was no significant change in MM (*p* = 0.3) Table 2.

### 3.5. Mouthwashes Intergroup Analysis

**Figure 2:**
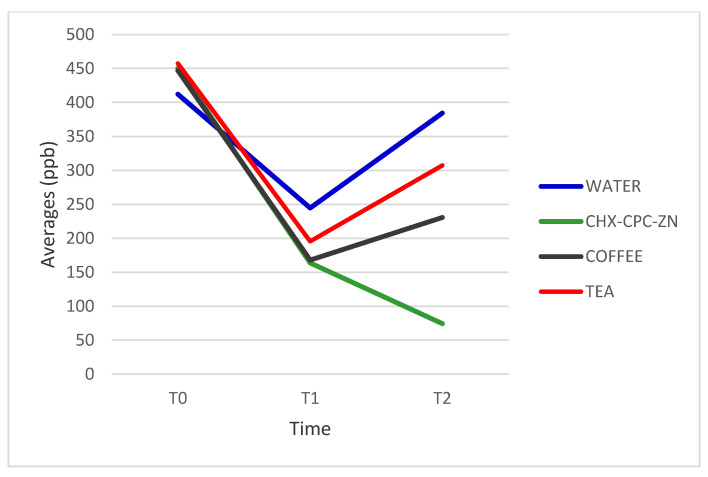
Variation in averages of H_2_S for all interventions at three time intervals.

**Figure 3:**
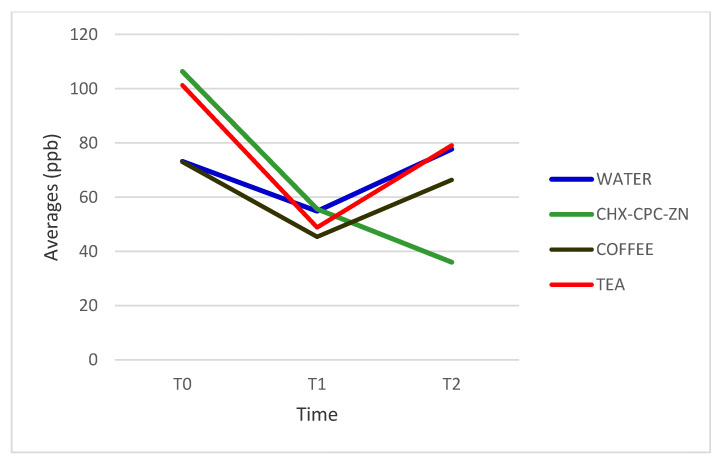
Variation in averages of methyl mercaptan for all interventions at three time intervals.

At baseline and the first hour, there was no significant difference in the means of H_2_S and MM in the pre-treatment breath samples of all four interventions (Figure 2 and Figure 3). On the contrary, there was a statistically significant difference in the means of H_2_S and MM at 2 h post-treatment among all four interventions (*p* = 0.000 and *p* = 0.034, respectively) (Table 3).

## 4. Discussion

All over the world, coffee and tea are the most consumed beverages after water [29]. Customarily, coffee and tea are used, especially in the morning, to reduce oral halitosis [14]. The present study aimed to assess the short-term effect of coffee and black tea on halitosis.

This study used a crossover trial technique, where each individual acts as his/her own control. The crossover study design decreased the chance of inter-individual variations that may influence the study outcome. On the contrary, the parallel design has a much greater inter-subject variation. Furthermore, a crossover study design is inherently more powerful than a parallel design for the same number of subjects recruited. An appropriate washout period is required for any crossover study to avoid any treatment intervention’s carry-over effects. The appropriate washout period for products would depend on their efficacy and/or their mode of action. For regular oral care products, a one-week washout period is considered an appropriate timescale to ensure no carry-over effects [30].

Several clinical trials have been conducted using either short- or long-term models to evaluate mouthwashes’ efficacy in reducing oral malodor. The long-term model is used to evaluate the therapeutic effect of using a particular mouthwash for a long period (2–3 weeks). On the other hand, the short-term model is used to assess the mouthwash’s immediate effect (masking) over 1–5 h. The cysteine challenge model was adopted in this study to evaluate the effect of various mouthwashes on halitosis [28]. Rinsing with cysteine (10 mL of 6 mM for 30 s) will raise the VSC production by the bacteria on the tongue and teeth, which will result in oral malodor. Cysteine breakdown occurs by oral bacteria into VSC, which can be assessed as a quantitative parameter using chromatography devices such as OralChroma [28].

The current study revealed an initial decrease in the VSC level during the first hour in both black tea and coffee groups. The decrease was followed by an increase in VSC concentration during the second hour, which indicates that coffee and black tea have no long-term effect on VSC reduction after 2 h. The current study results showed a statistically significant reduction in the VSC levels 2 h after using coffee. Gov et al. demonstrated in an in vitro study that coffee components significantly reduced VSC levels and VSC-producing bacteria [17]. It has been reported in several studies that coffee extracts contain substances that have both antimicrobial and antioxidant activities [16,31,32,33]. Roasted coffee’s antibacterial activity was attributable to the presence of compounds like caffeine, α-dicarbonyl, glyoxal, methylglyoxal, and diacetyl [34]. More specifically, in vitro studies have demonstrated that coffee extract has antibacterial effects against *P. gingivalis*, and *Prevotella intermedia*, which are the bacteria closely associated with halitosis [16,35,36]. Furthermore, the coffee extract can inhibit protease production by *P. gingivalis* [16]. Cardamom (*Elettaria cardamomum*), which is a traditional aromatic plant, is one of the major ingredients of Arabic coffee. It has been reported that cardamom has some antibacterial activities [37,38]. More specifically, Souiss et al. reported that cardamom inhibits the growth of periodontal pathogens such as: *P. gingivalis, P. intermedia,* and *Aggregatibacter actinomycetemcomitans;* those bacteria are highly associated with production of halitosis [38]. In a clinical trial, Erawati et al. stated that rinsing with a mouthwash containing 0.5% cardamom essential oil had reduced the levels of VSC in breath for up 5 h after rinsing [39].

The effect of green tea extract mouthwash on VSC was studied by Farina et al.; they observed that green tea had an immediate inhibitory effect on the production of VSC, with no residual inhibitory effects at 90 and 180 min. This is in agreement with the present study [40]. The black tea used in this study has a different composition from green tea due to the fermentation process, which degrades the phenolic compounds responsible for antibacterial and antioxidant activities [20,21]. Furthermore, the concentration of zinc in black tea is less than that in green tea [19]. The presence of zinc plays an important role in masking halitosis by binding to volatile H_2_S and MM, which will convert them into Zn-sulfides (non-volatile), reducing the expression of the VSCs in the breath [41].

In this study, the data demonstrated the positive impact of using CHX-CPC-Zn mouthwash on reducing the VSC. This study’s results are in agreement with those of previous studies, which showed the beneficial impact of using CHX-CPC-Zn mouthwash [13,42,43,44]. In a systematic review, Blom et al. concluded that CHX-CPC-Zn mouthwash provided the best evidence in the bifacial effect concerning halitosis [12].

Female subjects were not included in this trial due to fluctuations in sex hormone levels during the menstrual cycle, which will affect the levels of volatile sulfur compounds and thus affecting the outcome of the clinical trial [45,46].

One of the study’s limitations is the difficulty of blinding well-known drinks such as tea and coffee. The examiners were completely blinded; however, the participants could identify the type of drink they were taking. Future studies with larger sample size should be conducted.

## 5. Conclusions

This study demonstrates that tea and Arabic coffee had inhibitory effects on halitosis. The greater reduction in halitosis occurred in the first hour and was not sustained over a long period of time. Additionally, Arabic coffee had a greater inhibitory effect on halitosis than black tea.

## Figures and Tables

**Table 1:** Subjects’ characteristics.

Variables	Mean ± SD
Age	22.2(±4.1)
PI	1.05 (±0.77)
GI	1.26 (±0.74)
PD	2.1 (±0.92)

**Table 2:** Comparison of averages at three different times for each intervention.

Intervention	Mean (SD)			*p*-Value
	To	T1	T2	
	**H_2_S**			
WATER	412.18 (148.53)	244.54 (95.28)	384.36 (161.89)	0.710
CHX-CPC-ZN	450.54 (291.35)	163.72 (178.95)	74.27 (72.21)	0.000 *
COFFEE	446.82 (169.65)	167.82 (88.41)	230.54 (128.82)	0.000 *
TEA	457.27 (334.58)	195.54 (154.27)	307.27 (252.08)	0.045 *
	**Methyl mercaptan**			
WATER	73.18 (44.61)	54.82 (20.65)	77.72 (38.96)	0.306
CHX-CPC-ZN	106.36 (63.73)	55.63 (38.87)	36.0 (36.05)	0.005 *
COFFEE	73.0 (58.88)	45.36 (52.87)	66.36 (44.77)	0.445
TEA	101.27 (67.35)	48.82 (26.35)	79.09 (27.03)	0.033 *

* Statistically significant at the 0.05 significance threshold.

**Table 3:** Comparison of averages from four different interventions at given time.

Time	Mean (SD)				*p*-Value
	WATER	CHX-CPC-ZN	COFFEE	TEA	
	**H_2_S**				
BASELINE	412.18 (148.53)	450.54 (291.35)	446.82 (169.65)	457.27 (334.58)	0.975
T1	244.54 (95.28)	163.72 (178.95)	167.82 (88.41)	195.54 (154.27)	0.481
T2	384.36 (161.89)	74.27 (72.21)	230.54 (128.82)	307.27(252.08)	0.000 *
	**Methyl mercaptan**				
T0	73.18 (44.61)	106.36 (63.73)	73.0 (58.88)	101.27 (67.35)	0.403
T1	54.82 (20.65)	55.63 (38.87)	45.36 (52.87)	48.82 (26.35)	0.899
T2	77.72 (38.96)	36.0 (36.05)	66.36 (44.77)	79.09 (27.03)	0.034 *

* Statistically significant at the 0.05 significance threshold.

## Data Availability

No new data were created or analyzed in this study. Data sharing is not applicable to this article.
